# Rethinking Head Computed Tomography (CT) in the Emergency Department: From Reflex Imaging to Reasoned Care

**DOI:** 10.7759/cureus.103861

**Published:** 2026-02-18

**Authors:** So Sakamoto

**Affiliations:** 1 Emergency Medicine, Asahi General Hospital, Asahi, JPN

**Keywords:** canadian ct head rule, discharge planning, falls, head ct, mild traumatic brain injury (mtbi), older adults, shared decision-making

## Abstract

Head computed tomography (CT) is among the most frequently ordered tests in emergency care, yet its use for mild traumatic brain injury (mTBI) remains highly variable. Contemporary practice patterns reflect this: head CT is obtained in >80% of emergency department (ED) evaluations for suspected mTBI, and in older adults presenting after a fall, approximately half undergo cranial CT in routine practice (49% in a UK ED cohort aged ≥65 years). Ageing societies bring more older fallers to the ED, often with incomplete histories and widespread use of anticoagulants or antiplatelets. These realities lower imaging thresholds and can turn CT into a default substitute for clinical reasoning. This editorial argues for a reframing: head CT should be positioned as a decision-support tool within a broader diagnostic strategy, not as the endpoint of thinking. We highlight recurrent pitfalls: misunderstanding what decision rules, such as the Canadian CT Head Rule (CCHR), are designed to predict; applying CCHR outside its intended population; equating “any radiographic abnormality” with clinically important injury; and underestimating the diagnostic and therapeutic value of time, serial neurologic assessment, and high-quality discharge planning. Integrating evidence from decision rules, observational data in older fallers, and qualitative research on physician decision-making, we propose a practical approach: align imaging with the outcome of interest, respect rule assumptions and exclusions, and deliberately “use time” when immediate imaging is unlikely to change management. Ultimately, reducing unnecessary CT is not merely an educational problem; it requires a sustainable clinical ecosystem that supports judgment under uncertainty.

## Editorial

Head computed tomography (CT) is fast, familiar, and often accessible. It also carries an emotional promise: certainty. In the emergency department (ED), where time pressure and diagnostic uncertainty are constant, imaging can quietly become the default response to anxiety: clinician anxiety, patient anxiety, and system anxiety. The result is not simply “more CT,” but a subtle shift in practice: CT becomes the thinking, rather than a tool used after thinking. This drift is reflected in contemporary utilization: head CT is obtained in >80% of ED evaluations for suspected mTBI, and in older adults presenting after a fall, approximately half undergo cranial CT in routine practice [1,2].

In mTBI, the clinically important outcome is not “any abnormality on CT.” Outcomes that matter are those that change management and trajectory, especially neurosurgical intervention and clinically important brain injury (CIBI). Decision rules were built to protect patients from missing these high-stakes injuries while reducing low-yield imaging. When we lose sight of the intended outcome, we start optimizing for the wrong target: the elimination of all radiographic uncertainty.

CCHR is often treated as a universal detector of intracranial hemorrhage. It is not. CCHR was developed to identify patients at risk of neurosurgical intervention and CIBI while safely reducing unnecessary CT use [3]. Importantly, “clinically important” in the original CCHR framework is not synonymous with “any CT finding”; small lesions that do not require admission and neurologic follow-up were explicitly categorized as clinically unimportant [3]. This distinction matters because conflating “positive CT” with “important injury” pushes practice toward an impossible goal-perfect radiographic certainty-at the expense of interventions that actually influence outcomes: serial examination, safe disposition decisions, and coherent follow-up plans.

A second, underappreciated misuse is applying CCHR to “head trauma” broadly. CCHR was derived for patients with minor head injury [Glasgow Coma Scale (GCS) 13-15] who had witnessed loss of consciousness, definite amnesia, or disorientation [3]. Patients without these features-often described as having “minimal head injury”-were outside the original scope [3]. Yet in practice, especially among older fallers with unwitnessed events or unreliable histories, CCHR can be inadvertently repurposed as a general gatekeeper for CT. This drift creates two opposing hazards. First, clinicians may overtrust “rule negativity” in patients who were never eligible for the rule, creating a false sense of safety. Second, clinicians may overemphasize a single criterion (most commonly age) and scan nearly everyone, inflating CT use without meaningfully improving outcomes.

CCHR performance also depends on its assumptions and exclusions. In the derivation study, patients with bleeding disorders and those using oral anticoagulants (warfarin at the time) were excluded [3]. These exclusions do not imply that anticoagulated patients must always undergo CT; rather, they signal that mechanical “rule application” is inappropriate and that individualized judgment-grounded in symptoms, examination, mechanism, and feasible follow-up-is required [3].

Age is an important risk factor in head injury, but it can be misused as an automatic trigger. In real practice, the age criterion is frequently the sole reason for scanning otherwise low-risk patients, particularly older fallers with normal examinations. Fournier and colleagues explored whether raising the CCHR age cutoff (from 65 to 75 years) could reduce scanning while preserving safety; they suggested it could be safe, but emphasized the need for prospective validation [4]. The larger, pragmatic message is stable: age alone is rarely a sufficient argument for immediate CT. Age must be integrated with symptoms, physical examination, mechanism, medication profile, and, critically, the feasibility of safe observation and follow-up.

**Figure 1:**
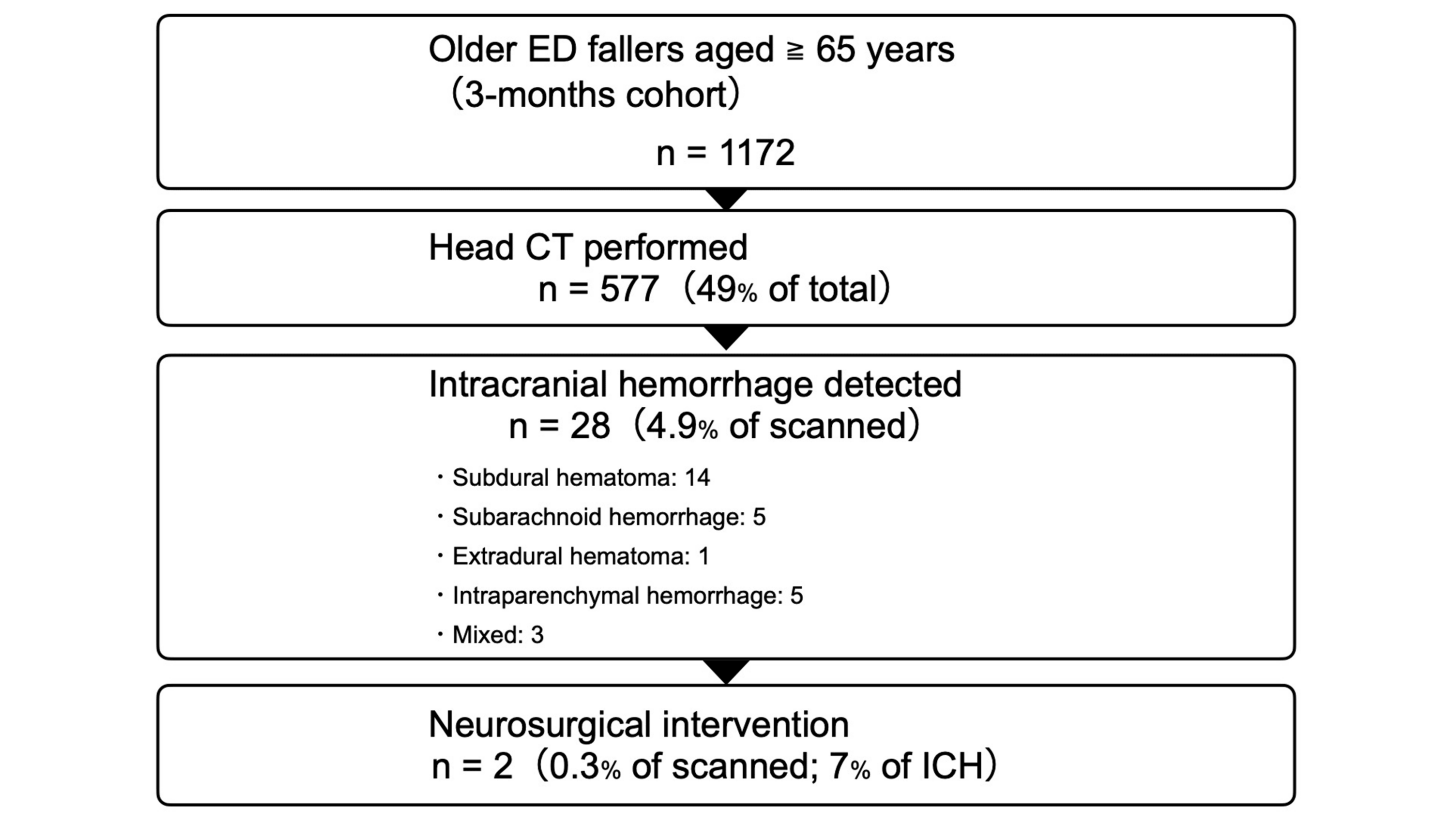
Head CT utilization and downstream yield in older ED fallers (UK cohort aged ≥65 years) In a UK emergency department (ED) cohort of fallers aged ≥65 years (N=1172), 49% underwent head CT (577/1,172). Intracranial hemorrhage was detected in 4.9% of those scanned (28/577), including subdural hematoma (14), subarachnoid hemorrhage (5), extradural hematoma (1), intraparenchymal hemorrhage (5), and mixed hemorrhage (3). Neurosurgical intervention occurred in 0.3% of those scanned (2/577; 7% of ICH). Data from Warren et al. [2].

A central “rethinking” insight is the gap between detecting abnormalities and changing outcomes. In older adults presenting after a fall, cranial CT is frequently obtained, yet neurosurgical intervention is rare. For example, in a UK ED cohort of fallers aged 65 years or older, nearly half received head CT; intracranial hemorrhage was identified in a small minority, and neurosurgical intervention occurred in only a tiny fraction [2]. A simple visual summary of this utilization-to-intervention drop-off is shown in Figure 1.

This does not argue against imaging; it clarifies what imaging often does in this population. CT commonly reassures, stratifies risk, and supports disposition decisions more than it triggers operative management. If we aim to prevent catastrophic misses, the key question becomes: which patients truly benefit from immediate CT, and for whom is a structured “time and observation” strategy the safer and more proportionate response?

Anticoagulant and antiplatelet use is common among older ED patients with head trauma, understandably lowering the imaging threshold. However, a medication-only reflex can drive over-imaging, especially when neurologic assessment is normal, and there are no high-risk clinical features. Davey and colleagues studied a cohort with minimal head injury who underwent CT; intracranial hemorrhage was rare, and the hemorrhages identified fell into higher-risk CCHR categories [5]. These findings should not be overinterpreted as “validation” of CCHR in anticoagulated patients or as proof that CT can always be avoided. Rather, they support a broader point: indiscriminate scanning can often be replaced by structured clinical features aligned with meaningful outcomes, provided we remain honest about rule scope and uncertainty.

If CT overuse were simply ignorance of decision rules, education would have solved it. Qualitative work suggests the real drivers are multi-layered: system context, staffing and support, professional responsibility, the reliability of patient information, and the goal of patient-centered care [6]. In addition, fear of litigation and defensive medicine can further lower the threshold for CT ordering, particularly when clinical uncertainty intersects with limited time, imperfect follow-up, and high perceived stakes. These pressures are amplified in older adults with cognitive impairment, polypharmacy, unwitnessed events, and competing medical risks. Therefore, reducing low-yield CT requires more than reminding clinicians about rules; it requires an ecosystem that makes high-quality observation, communication, and follow-up feasible.

Time is an underused diagnostic and therapeutic tool. Clinicians already “use time” after a negative CT by providing return precautions and anticipating delayed complications. For carefully selected low-risk patients, similar safety can be achieved by deliberately using time before CT through serial neurologic assessment, symptom evolution, and robust discharge planning, when immediate imaging is unlikely to change management.

Discharge communication is not a trivial afterthought. In a systematic review and meta-analysis, correct recall of discharge instructions was, on average, 47% with verbal instructions alone, 58% with printed materials, and 67% with video-based instructions [7]. This is not merely a communication statistic; it is a systems problem. “Using time” safely depends on whether patients and families understand the baseline, recognize warning signs, and know precisely when and how to return.

This is where shared decision-making becomes practical rather than aspirational. When immediate CT is unlikely to change management, clinicians can explicitly align the diagnostic strategy with outcomes that matter: preventing missed clinically important injury, maintaining functional safety, and minimizing iatrogenic harm from cascades triggered by incidental findings. Explaining what CT can and cannot guarantee-and what observation and follow-up are designed to achieve- allows patients and families to participate in a strategy that is both safer and more proportionate than reflex imaging.

Definitions also matter. Internationally, mTBI is often defined as GCS 13-15, yet this category is clinically heterogeneous. Contemporary policy highlights that patients with GCS 13 behave as a higher-risk subgroup; for example, the 2023 American College of Emergency Physicians clinical policy focuses its key recommendations on adults with GCS 14-15 and does not treat GCS 13 as mTBI for the purposes of its guidance [8]. Aligning terminology with risk, rather than tradition, helps prevent both under- and over-imaging and reminds clinicians that “mild” is not synonymous with “safe.”

Epidemiology reinforces why this matters. In the Japan Trauma Data Bank (2004-2019), older adults (≥65 years) accounted for 54.1% of TBI cases (50,990/94,180), and their share increased by 2.1% per year, outpacing the 0.5% annual increase in the general population [9]. Head CT decision-making in older fallers is therefore not a niche issue but a central one in contemporary emergency care.

Emerging work on blood-based biomarkers and advanced imaging may eventually refine diagnosis and prognosis in mTBI, particularly when CT is normal, yet symptoms persist. For example, glial fibrillary acidic protein (GFAP), ubiquitin C-terminal hydrolase-L1 (UCH-L1), and S100B have been studied as tools to help identify patients at low risk of intracranial injury and to support selective CT use or safe observation pathways. For now, routine ED implementation remains limited by access, timing, and clinical integration. These tools belong in a future layer of mTBI care, not as an excuse to bypass today’s essential work: thoughtful risk assessment, communication, and follow-up.

Rethinking head CT is not an argument against imaging. It is an argument for aligning imaging with outcomes that matter, respecting the assumptions, exclusions, and intended population of decision rules, and restoring time, serial examination, and discharge planning as core interventions. In older fallers and in patients taking anticoagulants or antiplatelets, CT thresholds are understandably lower, but reflexive scanning is not the only safe strategy. A sustainable clinical ecosystem-one that supports judgment under uncertainty-will reduce low-yield CT without increasing missed clinically important injury.
